# *MRE11A* Polymorphisms Are Associated With Subclinical Atherosclerosis and Cardiovascular Risk Factors. A Case-Control Study of the GEA Mexican Project

**DOI:** 10.3389/fgene.2019.00530

**Published:** 2019-05-31

**Authors:** Gilberto Vargas-Alarcón, Nonanzit Pérez-Hernández, José Manuel Rodríguez-Pérez, José Manuel Fragoso, Guillermo Cardoso-Saldaña, Christian Vázquez-Vázquez, Julian Ramírez-Bello, Carlos Posadas-Romero, Rosalinda Posadas-Sánchez

**Affiliations:** ^1^Department of Molecular Biology, Instituto Nacional de Cardiología Ignacio Chávez, Mexico City, Mexico; ^2^Department of Endocrinology, Instituto Nacional de Cardiología Ignacio Chávez, Mexico City, Mexico; ^3^Research Unit on Endocrine and Metabolic Diseases, Hospital Juárez de México, Mexico City, Mexico

**Keywords:** cardiovascular risk factors, DNA damage, meiotic recombination 11 homolog A, polymorphisms, subclinical atherosclerosis

## Abstract

DNA damage and subsequent repair pathways have been involved in the initiation and progression of atherosclerosis. Meiotic recombination 11 homolog A (*MRE11A*) gene polymorphisms have been associated with the presence of myocardial infarction. We analyzed five *MRE11A* gene polymorphisms in 386 individuals with subclinical atherosclerosis and 1093 healthy controls. Under different models, the rs13447720 (Odds ratio = 0.646, P_additive_ = 0.009; Odds ratio = 0.636, P_dominant_ = 0.012; Odds ratio = 0.664, P_over–dominant_ = 0.025; Odds ratio = 0.655, P_codominant1_ = 0.021) and rs499952 (Odds ratio = 0.807, P_additive_ = 0.032; Odds ratio = 0.643, P_codominant2_ = 0.034) polymorphisms were associated with a lower risk of subclinical atherosclerosis. On the other hand, the rs2155209 polymorphism was associated with a reduced risk of having a coronary artery calcification score ≥ 100 Agatston units. The rs13447720, rs499952, and rs2155209 polymorphisms, as well as the haplotypes that included the five studied polymorphisms were associated with some clinical and metabolic parameters in both subclinical atherosclerosis and healthy individuals. Our results suggest that the rs13447720 and rs499952 polymorphisms are associated with a decreased risk of developing subclinical atherosclerosis, whereas the rs2155209 is associated with a lower subclinical atherosclerosis severity (coronary artery calcification < 100 Agatston units). *MRE11A* polymorphisms and haplotypes were associated with clinical and metabolic parameters.

## Introduction

Atherosclerosis is a progressive and multifactorial disease conditioned by genetic and environmental factors and characterized by the accumulation of lipids in the intima and middle arterial layers. The clinical consequences of atherosclerosis constitute the underlying pathological process in myocardial ischemia, including coronary artery disease (CAD). Accumulated deoxyribonucleic acid (DNA) damage and subsequent repair pathways are now increasingly recognized as a causal factor for the genesis and progression of atherosclerosis (Shah and Mahmoudi, 2015). Two important causes of DNA damage are the oxidative stress and cigarette smoking (Corral-Debrinski et al., 1991; Andreassi, 2003). It has been reported that oxidative stress decreases anti-oxidant level and reduced DNA repair efficiency, and as a consequence, it could contribute to the progression of atherosclerotic coronary disease (Simon et al., 2013). Furthermore, tobacco components can produce oxidative stress that is accompanied by inflammation and apoptosis (Panda and Chatterjee, 2007; Banerjee et al., 2008). DNA damage associated with atherosclerosis occurs in circulating cells, vascular smooth muscle cells (VSMCs) and endothelial cells of the vessel wall. Unrepaired DNA damage promotes endothelial cell dysfunction, monocyte migration, macrophage and foam cell death, VSMC senescence, and eventually contributes to plaque rupture by inducing expression of adhesion molecules and release of inflammatory cytokines (Mahmoudi et al., 2006; Rodier et al., 2009; Uryga et al., 2016). A recent genome-wide association study analyzed common genetic variants in five DNA repair pathways in two independent cohorts (Verschuren et al., 2013). The results showed the association of the rs2155209 polymorphism of the meiotic recombination 11 homolog A (*MRE11A*) gene with the presence of myocardial infarction (Verschuren et al., 2013). This study evaluated the association of these polymorphisms with a severe state of atherosclerosis (myocardial infarction); hence, we decided to evaluate the role of the same polymorphisms in early-stage atherosclerosis. Thus, the aim of the present study was to analyze the distribution of *MRE11A* polymorphisms in asymptomatic individuals with subclinical atherosclerosis (SA)–defined as a coronary artery calcification (CAC) score greater than zero–in order to establish its role in early atherosclerosis. For our study, we selected the five polymorphisms which had been previously analyzed in individuals with myocardial infarction in the genome-wide association study (Verschuren et al., 2013).

## Materials and Methods

### Subjects

The present study included 1479 apparently healthy asymptomatic individuals without personal or family history of premature CAD, recruited from blood bank donors and through brochures posted in social services centers. These individuals belong to the GEA Mexican Study, which was approved by Bioethics and Investigation Committee of the Instituto Nacional de Cardiología Ignacio Chávez (INCICH, Project number 09-646). All participants provided informed consent. The study was conducted in accordance with the Helsinki Declaration. Exclusion criteria included congestive heart failure and liver, renal, thyroid or oncological disease. Standardized questionnaires were applied to all participants to obtain demographic information, history of nutritional habits, physical activity, family medical history, alcohol consumption and pharmacological treatment. Demographic, clinical, anthropometric, biochemical parameters and cardiovascular risk factors were evaluated in all individuals as previously described (Medina-Urrutia et al., 2015; Posadas-Sánchez et al., 2017a,b).

### Computed Axial Tomography Study

The following parameters were determined in all individuals by a computed tomography of the chest and abdomen using a 64-channel multi-detector helical computed tomography system (Somatom Sensation, Siemens): CAC score using the Agatston method (Mautner et al., 1994), total abdominal fat (TAF), subcutaneous and visceral abdominal fat (SAF and VAF) areas as described by Kvist et al. (1988), and hepatic to splenic attenuation ratio as reported by Longo et al. (1993). Of the 1479 individuals included in the study, 386 had a CAC score greater than 0 and were considered SA individuals, whereas 1093 subjects had a CAC score of zero and hence conformed the group of healthy controls.

### Genetic Analysis

High-molecular-weight genomic DNA was extracted from peripheral blood using the QIAamp DNA Blood Mini kit (QIAGEN, Hilden, Germany). According to the manufacturer’s instructions (Applied Biosystems, Foster City, CA, United States), the rs2155209 (C___2020632_20), rs535801 (C___1000978_10), rs529126 (C___2020532_10), rs499952 (C__26487289_10), and rs13447720 (C__33306696_10) *MRE11A* polymorphisms were determined in genomic DNA using 5′ exonuclease TaqMan genotyping assays on an ABI Prism 7900HT Fast Real-Time PCR system. Previously sequenced samples of the different polymorphisms studied were included as controls. The controls cover all expected genotypes for the polymorphisms tested. In order to corroborate the adequate assignment of the genotypes in the TaqMan assays, we randomly select and repeat 10% of the samples. These samples were 100% concordant in two independent assays. However, it is important to note that it cannot be excluded that the homozygous wild type and mutant genotyping results were negatively affected by allele drop-out events.

### Statistical Analysis

Data are expressed as the mean (standard deviation), median (interquartile range) or frequencies. Analysis of continuous and categorical variables was made using Student t, Mann–Whitney *U* and chi-square tests, as appropriate. Associations of polymorphisms with SA, cardiometabolic parameters and cardiovascular risk factors under different inheritance models (additive, co-dominant 1, co-dominant 2, dominant, over-dominant, and recessive) were evaluated using logistic regression analysis. When the association with SA was tested, the models were adjusted for age, gender, body mass index (BMI), current smoking, hypertension, type 2 diabetes mellitus (T2DM), and low-density lipoprotein cholesterol (LDL-C). To evaluate the association with cardiovascular risk factors, the models were adjusted for age, gender and BMI. Models were constructed using one variable at a time; final models included variables with biological relevance or with statistical significance, or both. All the polymorphisms studied were in Hardy–Weinberg equilibrium (*P* > 0.05). We used SPSS software v15.0 (SPSS Chicago, IL, United States) for all analyses. Pair wise linkage disequilibrium (LD, D′) and haplotype construction were performed with Haploview version 4:1 (Broad Institute of Massachusetts Institute of Technology and Harvard University, Cambridge, MA, United States). A value of *P* < 0.05 was considered significant.

## Results

### Clinical, Metabolic, and Cardiovascular Risk Factors

Clinical and metabolic characteristics of the studied groups are shown in Table 1. Compared with the healthy control group, SA individuals had higher values of waist circumference, systolic and diastolic blood pressure, glucose, triglycerides, and total, low-density lipoprotein (LDL) and non-HDL cholesterol. As shown in Table 2, the prevalence of hypercholesterolemia, hypertriglyceridemia, total adipose fat (TAF) > p75, T2DM, hyperinsulinemia and hypertension were higher in SA individuals when compared with controls.

**Table 1:** Clinical and metabolic characteristics of the studied groups.

	Control (*n* = 1093)	Subclinical atherosclerosis (*n* = 386)	*P*
Age (years)	51 ± 9	59 ± 8	<0.0001
Gender (% male)	40.9	75.4	<0.0001
Body mass index (kg/m^2^)	27.9 [25.4–31.0]	28.1 [25.9–31.0]	0.127
Waist circumference (cm)	93.6 ± 11.2	97.2 ± 10.6	<0.0001
Systolic blood pressure (mmHg)	112 [104–123]	122 [111–133]	<0.0001
Diastolic blood pressure (mmHg)	70 [65–77]	74 [68–82]	<0.0001
Total abdominal fat (cm^2^)	432 [347–536]	446 [354–556]	0.108
Total cholesterol (mg/dL)	190 [166–210]	198 [169–220]	0.002
High density lipoprotein cholesterol (mg/dL)	45 [36–55]	43 [36–51]	0.023
Low density lipoprotein cholesterol (mg/dL)	115 [95–134]	124 [102–145]	<0.0001
Triglycerides (mg/dL)	145 [108–202]	156 [118–205]	0.03
Non-HDL-cholesterol (mg/dL)	142 [121–163]	153 [128–175]	<0.0001
Glucose (mg/dL)	90 [84–97]	94 [86–105]	<0.0001
Insulin (μUI/mL)	17 [12–23]	18 [13–24]	0.101

*HDL, high density lipoprotein. Data are shown as mean ± standard deviation o median [interquartile range]. Student t-test, Mann-Whitney U or Chi-square test.*

**Table 2:** Cardiovascular risk factors prevalence in the study population.

	Control (*n* = 1093)	Subclinical atherosclerosis (*n* = 386)	^∗^*P*
Hypercholesterolemia (%)	29.6	43.5	<0.0001
Hypoalphalipoproteinemia (%)	51.9	44.6	0.008
Hypertriglyceridemia (%)	47.1	52.3	0.044
LDL pattern B	47.1	47.0	0.518
Gamma glutamyl transpeptidase activity > p75 (%)	41.4	44.3	0.176
Central obesity (%)	81.1	82.9	0.245
Total abdominal fat (%)	53.0	64.5	<0.0001
Type 2 diabetes mellitus (%)	9.7	22.3	<0.0001
Hyperinsulinemia (%)	52.3	61.1	0.002
Hypertension (%)	19.1	38.3	<0.0001
Vitamin D deficiency (%)	88.2	89.6	0.260
Current smoking (%)	22.7	21.8	0.382

*Data is shown as percentage. LDL, low density lipoprotein. Hypercholesterolemia was defined as low density lipoprotein cholesterol concentrations ≥ 130 mg/dL. ^∗^Chi-square test.*

### Associations With SA, Metabolic, and Cardiovascular Risk Factors

Two out of five study polymorphisms had different distributions in SA individuals (CAC > 0) and healthy controls (CAC = 0) (Table 3). Under different models, adjusted for age, gender, BMI, current smoking, hypertension, T2DM, and LDL-C concentrations, the rs13447720 *C* and rs499952 *G* alleles were associated with a decreased risk of developing SA.

**Table 3:** Association between rs13447720 and rs499952 *MRE11A* gene polymorphisms and subclinical atherosclerosis.

Polymorphism	Genotype frequency			Allele frequency	Model	OR [95% CI]	*P*
rs13447720	*TT*	*TC*	*CC*	*T/C*			
Control (*n* = 1093)	0.780	0.206	0.015	0.882/0.118	Additive	0.646 [0.465–0.897]	0.009
					Dominant	0.636 [0.447–0.905]	0.012
SA (*n* = 386)	0.824	0.168	0.008	0.908/0.092	Recessive	0.401 [0.093–1.722]	0.219
					Over-dominant	0.664 [0.463–0.951]	0.025
					Codominant1	0.655 [0.457–0.939]	0.021
					Codominant2	0.370 [0.086–1.596]	0.183

**rs499952**	***GG***	***GT***	***TT***	***G/T***			

Control (*n* = 1093)	0.414	0.457	0.129	0.642/0.358	Additive	0.807 [0.664–0.981]	0.032
					Dominant	0.709 [0.486–1.034]	0.074
SA (*n* = 386)	0.381	0.456	0.163	0.609/0.391	Recessive	0.777 [0.588–1.027]	0.076
					Over-dominant	1.073 [0.816–1.412]	0.617
					Codominant1	0.776 [0.519–1.162]	0.218
					Codominant2	0.643 [0.427–0.968]	0.034

*Models were adjusted for age, gender, body mass index, current smoking, hypertension, type 2 diabetes mellitus, low density lipoprotein cholesterol concentrations. OR, odds ratio; CI, confidence intervals; SA, subclinical atherosclerosis.*

**FIGURE 1:**
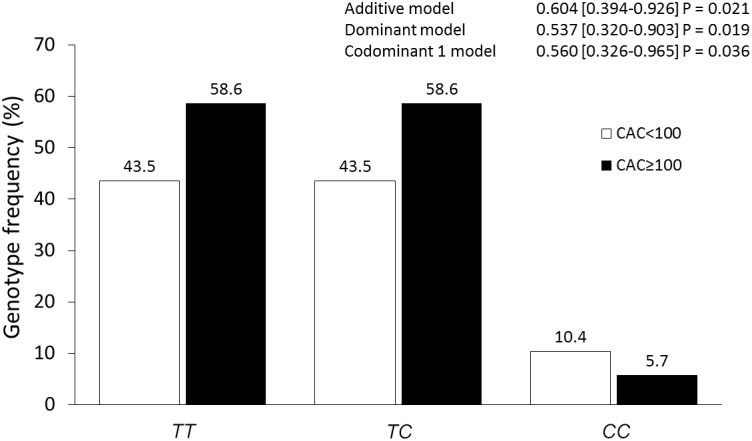
Association of the rs2155209 polymorphism with CAC severity. The models were adjusted for age, gender, body mass index, current smoking, hypertension, type 2 diabetes mellitus, low density lipoprotein cholesterol concentrations.

Previously, the MESA study has reported that the risk of coronary event increased 3.6, 7.73, and 9.67-fold in individuals with CAC 1–100, 101–300, and >300 Agatston units (AU), respectively. Therefore, we analyzed the association of the polymorphisms with CAC levels comparing those individuals with CAC < 100 AU versus individuals with CAC ≥ 100 AU. Under additive, dominant, and codominant1 models, after adjusting for age, gender, BMI, current smoking, hypertension, T2DM, and LDL-C concentrations, the rs2155209 polymorphism was associated with a decreased risk of having CAC ≥ 100 (Figure 1).

Association of the polymorphisms with metabolic and cardiovascular risk factors was evaluated independently in SA individuals and in healthy controls. In this analysis we included the polymorphisms that previously were detected associated with SA and CAC (rs13447720, rs499952, and rs2155209). In healthy controls under different models, the rs13447720 polymorphism was associated with a reduced risk of LDL pattern B, hyperinsulinemia, and hypertension, whereas the rs2155209 was associated with central obesity, LDL-C ≥ 130 mg/dL and vitamin D deficiency (Table 4). On the other hand, in SA individuals under different models, the rs13447720 polymorphism was associated with a raised risk of central obesity, and hypertension and with a diminished risk of high Gamma glutamyl transpeptidase (GGT) activity and vitamin D deficiency, whereas, the rs499952 polymorphism was associated with a decreased risk of high GGT activity. Finally, rs2155209 was associated with a heightened risk of central obesity (Table 5). All models were adjusted for age, gender and BMI.

**Table 4:** Association between *MRE11A* gene polymorphisms and metabolic abnormalities in the control group.

Polymorphism	Genotype frequency			Allele frequency	Model	OR [95% CI]	*P*
rs13447720	*TT*	*TC*	*CC*	*T/C*			
*LDL pattern B*							
No (*n* = 578)	0.750	0.231	0.019	0.866/0.134	Additive	0.710 [0.542–0.930]	0.013
Yes (*n* = 515)	0.812	0.178	0.010	0.901/0.099	Dominant	0.699 [0.520–0.940]	0.018
					Over-dominant	0.729 [0.539–0.987]	0.041
					Codominant1	0.719 [0.531–0.974]	0.033
*Hyperinsulinemia*							
No (*n* = 521)	0.752	0.231	0.017	0.868/0.132	Dominant	0.721 [0.522–0.996]	0.047
Yes (*n* = 572)	0.804	0.184	0.012	0.896/0.104			
*Hypertension*							
No (*n* = 884)	0.767	0.129	0.014	0.877/0.123	Dominant	0.626 [0.414–0.946]	0.026
Yes (*n* = 209)	0.833	0.148	0.019	0.907/0.093	Over-dominant	0.578 [0.375–0.889]	0.013
					Codominant1	0.528 [0.378–0.896]	0.014

**rs2155209**	***TT***	***TC***	***CC***	***T/C***			

*Central obesity*							
No (*n* = 207)	0.427	0.500	0.073	0.676/0.324	Additive	1.472 [1.045–2.073]	0.027
Yes (*n* = 886)	0.442	0.431	0.126	0.658/0.342	Recessive	3.055 [1.414–6.601]	0.004
					Codominant2	3.163 [1.403–7.133]	0.006
*LDL-cholesterol ≥ 130 mg/dL*							
No (*n* = 770)	0.458	0.424	0.118	0.670/0.330	Dominant	1.344 [1.030–1.754]	0.030
Yes (*n* = 323)	0.394	0.495	0.111	0.641/0.359	Over-dominant	1.390 [1.068–1.808]	0.014
					Codominant1	1.417 [1.071–1.874]	0.015
*Vitamin D deficiency*							
No (*n* = 129)	0.516	0.320	0.164	0.678/0.322	Dominant	1.436 [1.002–2.137]	0.049
Yes (*n* = 964)	0.429	0.460	0.111	0.660/0.340	Over-dominant	1.891 [1.265–2.827]	0.002
					Codominant1	1.805 [1.183–2.753]	0.006

*Models were adjusted for age, gender, and BMI. OR, odds ratio; CI, confidence intervals.*

**Table 5:** Association between *MRE11A* gene polymorphisms and metabolic abnormalities in subclinical atherosclerosis subjects.

Polymorphism	Genotype frequency			Allele frequency	Model	OR [95% CI]	*P*
rs13447720	*TT*	*TC*	*CC*	*T/C*			
*Central obesity*							
No (*n* = 66)	0.955	0.045	0	0.977/0.023	Additive	5.338 [1.305–21.84]	0.020
Yes (*n* = 320)	0.797	0.194	0.009	0.788/0.212	Dominant	5.491 [1.328–22.70]	0.019
					Over-dominant	5.115 [1.223–21.38]	0.025
					Codominant1	5.220 [1.248–21.83]	0.024
*GGT activity > p75*							
No (*n* = 215)	0.800	0.186	0.014	0.893/0.107	Additive	0.534 [0.311–0.917]	0.023
Yes (*n* = 171)	0.854	0.145	0	0.927/0.073	Dominant	0.552 [0.314–0.971]	0.039
*Hypertension*							
No (*n* = 238)	0.861	0.139	0	0.931/0.069	Additive	1.856 [1.088–3.166]	0.023
Yes (*n* = 148)	0.764	0.216	0.020	0.872/0.128	Dominant	1.793 [1.023–3.144]	0.041
*Vitamin D deficiency*							
No (*n* = 40)	0.825	0.125	0.050	0.888/0.112	Recessive	0.032 [0.002–0.419]	0.009
Yes (*n* = 346)	0.824	0.173	0.003	0.9110.089	Codominant2	0.034 [0.003–0.449]	0.010

**rs499952**	***CC***	***TC***	***TT***	***C/T***			

*GGT activity > p75*							
No (*n* = 215)	0.367	0.502	0.130	0.619/0.381	Codominant1	0.505 [0.277–0.921]	0.026
Yes (*n* = 171)	0.398	0.398	0.205	0.596/0.404			

**rs2155209**	***TT***	***TC***	***CC***	***T/C***			

*Central obesity*							
No (*n* = 66)	0.576	0.318	0.106	0.735/0.265	Dominant	2.368 [1.090–5.143]	0.029
Yes (*n* = 320)	0.447	0.463	0.091	0.678/0.322	Over-dominant	2.473 [1.103–5.543]	0.028

*Models were adjusted for age, gender, and BMI. OR, odds ratio; CI, confidence intervals.*

### Haplotype Analysis

The five studied polymorphisms were in high linkage disequilibrium (Supplementary Table 1). None of the haplotypes formed were associated with risk of developing SA (data not shown). However, some of the haplotypes were associated with risk of metabolic and cardiovascular risk factors in SA subjects and healthy controls (Table 6). In healthy controls, the *TTTGC* and *CTTTT* haplotypes were associated with a decreased risk of central obesity; the *TTTTT* haplotype was nominally associated with a reduced risk of hypercholesterolemia. Moreover, the *TCGGC* haplotype was associated with a diminished risk of LDL pattern B and the *TTGGT* haplotype showed a significant association with an increased risk of LDL pattern B and hyperinsulinemia. On the other hand, in SA subjects, the *TTGGT* haplotype was associated with decreased risk of central obesity, and the *TCGGC* haplotype was additionally associated with a raised risk of central obesity, hypertension and total abdominal fat (TAF) > p75.

**Table 6:** Association of *MRE11A* haplotypes with presence of cardiometabolic risk factors.

			Variable presence			
Group	Variable	Haplotype	Yes	No	OR [95% CI]	*P*
			**HF**	**HF**		
*Control*						
	Central obesity	*TTTGC*	0.090	0.125	0.694 [0.496–0.971]	0.033
		*CTTTT*	0.008	0.020	0.402 [0.168–0.996]	0.041
	Hypercholesterolemia	*TTTTT*	0.191	0.234	0.771 [0.613–0.971]	0.027
	LDL pattern B	*TTGGT*	0.197	0.163	1.261 [1.012–1.572]	0.039
		*TCGGC*	0.084	0.120	0.676 [0.509–0.898]	0.007
	Hyperinsulinemia	*TTGGT*	0.197	0.161	1.227 [1.023–1.593]	0.030
*Subclinical atherosclerosis*						
	Central obesity	*TTGGT*	0.164	0.252	0.582 [0.373–0.911]	0.018
		*TCGGC*	0.099	0.023	4.703 [1.454–15.21]	0.010
	Hypertension	*TCGGC*	0.126	0.061	2.211 [1.328–3.680]	0.002
	TAF > p75	*TCGGC*	0.102	0.057	1.962 [1.082–3.561]	0.027

*The order of the polymorphism in the haplotype is according to the position in the chromosome (rs2155209, rs13447720, rs499952, rs529126, and rs535801). Hypercholesterolemia was defined as LDL-C concentrations ≥ 130 mg/dL. OR, odds ratio; CI, confidence interval; HF, haplotype frequency, TAF, total abdominal fat.*

## Discussion

A growing body of evidence has suggested that cells present in the atherosclerotic plaque and in circulation of coronary patients show DNA damage, which correlates with disease severity (Botto et al., 2001; Demirbag et al., 2005). Smoking and diabetes, two important cardiovascular risk factors, have been shown to directly cause oxidative DNA damage and inhibition of DNA repair mechanisms (Basta et al., 2004). MRE11A is an important molecule which participates in the DNA repair mechanisms. In addition, MRE11A has double-stranded (ds) DNA exonuclease and single-stranded (ss) DNA endonuclease activity in both homologous recombination and non-homologous end-joining (Xie et al., 2009). Recently, a gene set analysis of GWAS has reported the association of the *MRE11A* rs2155209 polymorphism with the presence of myocardial infarction in two independent cohorts (Verschuren et al., 2013). In our study, where 1479 individuals (386 with SA and 1093 healthy controls) were analyzed, the association of this polymorphism with SA (defined as individuals with CAC > 0) was not detected. The fact that this polymorphism is associated with myocardial infarction but not with SA suggests that it could be a marker for severe disease. In order to explore this possibility, we analyzed the association of the *MRE11A* polymorphisms with CAC ≥ 100 AU –as a marker for severity. For this analysis, we separated our group of SA in individuals with low (<100 AU) and high (≥100 AU) CAC score. As a result, the rs2155209 polymorphism was associated with a decreased risk of having high CAC, suggesting that this polymorphism could be a protective marker for atherosclerosis severity. The differences observed can be due to selection inclusion criteria of the participants in each study. The study by Verschuren included patients with myocardial infarction. On the contrary, our study included individuals without clinical evidence of CAD but with the presence of CAC. Also, the participants of our study were apparently healthy asymptomatic individuals without personal or family history of premature CAD, recruited from blood bank donors and through brochures posted in social services centers. Thus, the individuals of our survey were different to the subjects included in the study of Verschuren et al. (2013). This polymorphism also showed a significant association with an increased risk of central obesity in both SA and healthy individuals and with a raised risk of hypercholesterolemia (defined as LDL-C ≥ 130 mg/dL) and vitamin D deficiency in healthy controls. The rs2155209 polymorphism is located in the 3′ UTR region of the gene and, according to the *in silico* analysis, produces a binding site for the microRNA 1296. It has been reported that this polymorphism is located in a DNase I hypersensitivity site that is associated with *cis*-regulatory sequences, such as promoters, insulators, enhancers and locus control regions (Verschuren et al., 2013). However, to the best of our knowledge, the functional effects of this polymorphism have not been investigated thus far.

Two other polymorphisms (rs13447720 and rs499952) were associated with a reduced risk of developing SA and with some cardiovascular risk factors. In healthy controls, the rs13447720 polymorphism was significantly associated with the presence of small and dense LDL particles (LDL pattern B), hyperinsulinemia, hypertension and vitamin D deficiency. Conversely, in the SA group, the association of this polymorphism with central obesity, GGT activity, hypertension and vitamin D deficiency was found. In addition, the rs499952 polymorphism was associated with GGT activity. According to the informatics tools, both polymorphisms are located in intronic regions and none of them were functional. However, it is important to consider that no experimental studies have defined the functionality of these polymorphisms yet. As far as we know, this is the first study to analyze the polymorphisms present in the *MRE11A* gene in SA. As commented before, a previous GWAS performed in two cohorts (GENDER- GENetic DEterminants of Restenosis) and (PROSPER- ROSPective study for the Elderly at Risk) reported the association of the *MRE11A* rs2155209 polymorphism with myocardial infarction (P_GENDER_ = 0.0032 and P_PROSPER_ = 0.0027) (Verschuren et al., 2013). Other studies have analyzed the association of polymorphisms located in genes that encode for molecules related with DNA repair in cardiovascular diseases. Some of these studies include the association of the Arg399Gln (rs25487) polymorphism in the XRCC1 base excision repair (*BER*) gene with stroke and coronary atherosclerosis (Mahabir et al., 2007; Bazo et al., 2011). In the same way, Shyu et al. (2012) reported the association of other genes related with DNA repair with large artery atherosclerotic stroke in smoking individuals.

It is important to notice that the association of the rs13447720 polymorphism with hypertension was different when SA individuals and controls were analyzed independently. This analysis was adjusted by gender, age and BMI. While the rs13447720 *C* allele was associated with a decreased risk of hypertension in healthy controls, in SA individuals, the same allele was associated with an increased risk of hypertension. As can be seen in Tables 1, 2, this apparently contradictory findings could be due to that fact that both groups have several metabolic and clinical parameters differences as a consequence of the stratification on the bases of the presence or not of CAC > 0. One healthy individual who carries the rs13447720 *C* allele could have low risk of developing hypertension, whereas an individual with SA (early-stage atherosclerosis) who carries this allele could have high risk of developing hypertension. Indeed, another difference between the study groups is the presence of coronary artery calcification in SA individuals, which is absent in the healthy controls. Only two studies have established a significant association of CAC with the incidence of hypertension (Peralta et al., 2010; Grossman et al., 2013). Nevertheless, this finding could explain the higher prevalence of hypertension in individuals with SA (38.3%) compared to healthy controls without CAC (19.1%). It has been reported that hypertensive subjects have higher DNA damage levels than the normotensive ones. Although no study has directly evaluated the effect of hypertension on DNA damage, a recent population study has shown that hypertension was the strongest determinant of oxidative stress in subjects with high cardiovascular disease risk (Fandos et al., 2009).

In both groups, the rs13447720 *C* allele was associated with high risk of vitamin D deficiency. The association of the vitamin D deficiency with cardiovascular disease is controversial with positive and negative associations. Shor et al. (2012) reported the association between vitamin D deficiency and high prevalence of CAD and cardiovascular events, such as myocardial infarction. In contrast, an association between cardiovascular disease and vitamin D deficiency was not found by de Boer et al. (2009) and Deleskog et al. (2012), which was corroborated by our research group in Mexican individuals with CAD (López-Bautista et al., 2017). It is well known that vitamin D levels are associated with lower levels of primary DNA damage. Taken together, these results suggest that there may be a relationship between the polymorphism rs13447720, vitamin D deficiency and DNA damage.

In our study, a haplotype analysis was included. The decision was made considering that the haplotypes may be in closer linkage disequilibrium with a causal variant than any single measured polymorphism, and therefore may enhance the coverage value of the genotypes over single polymorphism analysis. On the other hand, haplotypes may themselves be the causal variants of interest. With this strategy, we detected some haplotypes associated with metabolic parameters in both individuals with SA and healthy controls. Similar to what was detected in the polymorphism analysis, we observed haplotypes associated with LDL pattern B and hyperinsulinemia in healthy controls, and with central obesity and hypertension in SA individuals. These findings corroborate the association of the studied polymorphisms with the above-mentioned clinical parameters.

The study limitations should be considered. Firstly, we analyzed only five polymorphisms previously associated with myocardial infarction, and only one of these polymorphisms was functional according to informatics tools. Secondly, our study only included individuals with SA defined by CAC levels, and the analysis of these polymorphisms in patients with CAD is mandatory. Thirdly, due to the transversal character of the study, conclusions on causality cannot be made. Fourthly, considering that the selection of participants was not random, the findings may not be applicable to the general population. However, considering that the participants have no knowledge of their genotypes, their distribution would be expected to be similar in a randomly selected sample.

In summary, our results indicate that the rs13447720 and rs499952 polymorphisms are associated with a decreased risk of developing SA, whereas the rs2155209 is associated with a reduced risk of having CAC ≥ 100 AU. The three polymorphisms and haplotypes with the five studied polymorphisms showed association with some clinical and metabolic parameters. To the best of our knowledge, this is the first study to evaluate the association of the *MRE11A* polymorphisms with SA, clinical and metabolic parameters and CAC score. For this reason, the detected associations are not yet definitive, and replicate studies in independent populations are warranted to confirm these findings.

## Ethics Statement

All subjects gave written informed consent in accordance with the Declaration of Helsinki. The protocol was approved by the of Bioethics and Investigation Committee of the Instituto Nacional de Cardiología Ignacio Chávez (INCICH, Project number 09-646).

## Author Contributions

GV-A and RP-S contributed to conceptualization, acquired the funding, performed the project administration, and visualized the study. NP-H and JR-B performed the data curation. CV-V, JR-B, and RP-S performed the formal analysis. GV-A, JR-P, and JF investigated the data. NP-H, JR-P, JF, GC-S, and CV-V performed the methodology. GV-A supervised the study. GV-A, CP-R, and RP-S wrote the original draft of the manuscript, reviewed, and edited the manuscript. All authors contributed to manuscript revision, and read and approved the submitted version.

## Conflict of Interest Statement

The authors declare that the research was conducted in the absence of any commercial or financial relationships that could be construed as a potential conflict of interest.
