# Supplementary material for: MRE11A Polymorphisms Are Associated With Subclinical Atherosclerosis and Cardiovascular Risk Factors. A Case-Control Study of the GEA Mexican Project
Source: Front Genet. 2019 May 31;10:530. doi: 10.3389/fgene.2019.00530 (PMC6555271; doi:10.3389/fgene.2019.00530)
Supplement: Supplementary file 1 [file Table_1.DOCX]

Supplemental material

Table 1. Linkage disequilibrium analysis, D´and r2 values

| Polymorphism | | D | r^2^ |
| --- | --- | --- | --- |
| rs2155209 | rs13447720 | 1.0 | 0.062 |
| rs2155209 | rs499952 | 0.908 | 0.237 |
| rs2155209 | rs529126 | 0.883 | 0.138 |
| rs2155209 | rs535801 | 0.936 | 0.340 |
| rs13447720 | rs499952 | 0.866 | 0.054 |
| rs13447720 | rs529126 | 0.862 | 0.033 |
| rs13447720 | rs535801 | 0.842 | 0.069 |
| rs499952 | rs529126 | 0.957 | 0.565 |
| rs499952 | rs535801 | 0.445 | 0.147 |
| rs529126 | rs535801 | 0.886 | 0.358 |
